# A Retrospective Analysis of Length of Stay in Postoperative Fracture Repair Patients Receiving Patient-Controlled Analgesia Versus Nurse-Administered Analgesia

**DOI:** 10.5812/aapm-162394

**Published:** 2025-10-18

**Authors:** Sherry Luo, Joshua Nougaisse, Devaki Kalvapudi, Hardeep Singh, Jake Slaton

**Affiliations:** 1 Northeast Georgia Medical Center, Transitional Year, GA 30501, Gainesville, USA; 2 Department of GME Research and Quality Improvement, Northeast Georgia Medical Center, GA 30501, Gainesville, USA

**Keywords:** Analgesia, Patient-Controlled Analgesia, Postoperative Analgesia, Nurse-Administered Analgesia

## Abstract

**Background:**

Patient-controlled analgesia (PCA) is a widely used method for managing postoperative pain. However, its impact on hospital length of stay (LOS) is unclear due to patient population variation. Currently, there is limited data directly comparing LOS in limb fracture patients receiving PCA versus those exclusively receiving nurse-administered analgesia (NAA).

**Objectives:**

To assess the impact of PCA in combination with NAA on hospital LOS and postoperative pain scores in limb fracture surgery patients compared to NAA alone.

**Methods:**

A retrospective chart review was conducted to examine the LOS between all postoperative Northeast Georgia Health System (NGHS) patients between 18 and 75 years of age who underwent surgical limb fracture repairs between 2019 and 2024, specifically evaluating those who exclusively received NAA versus those who received a combination of PCA and NAA. The PCA and NAA groups each consisted of 49 patients. The PCA group self-administered intravenous (IV), epidural, or peripheral nerve analgesics via PCA pumps in addition to receiving nurse-administered transdermal or intramuscular analgesics. The NAA group received transdermal, intramuscular, or IV analgesics exclusively via manual administration by nursing staff. Medications included in this study were morphine, oxycodone, hydromorphone, fentanyl, and acetaminophen. Measured outcome variables include hospital LOS and pre- and postoperative pain scores, which were directly taken from NGHS’s electronic medical record.

**Results:**

The two groups studied included patients who received IV PCA in addition to NAA (termed as PCA) and patients who exclusively received transdermal, intramuscular, and/or IV NAA (termed as non-PCA). A total of n = 49 patients underwent limb fracture repair and received PCA, and 49 patients from the non-PCA group were matched accordingly. After propensity matching, average preoperative pain scores between the non-PCA and PCA groups were similar at 5.64 and 5.60, respectively. Patients in the PCA group had higher mean postoperative pain scores (μ = 4.92) compared to the NAA group (μ = 4.41), with a mean difference of 0.51 points (P = 0.046).

**Conclusions:**

This retrospective analysis suggests that the use of PCA in conjunction with NAA is associated with increased LOS and higher postoperative pain scores when compared to NAA alone in patients undergoing surgical repair of limb fractures.

## 1. Background

Fractures have become increasingly common in the past two decades, driven largely by population growth and aging. In the United States alone, approximately 9 million incident cases took place in 2019 (1). Patients in older age groups are more likely to have fractures; low-trauma fractures not only lead to an increased risk for refracture, but they also increase the risk for other types of clinical fractures (1, 2). As life expectancy rises, the burden of age-related falls is expected to grow in tandem (3). Adults more often require surgical management when compared with children (4). The average postoperative length of stay (LOS) in upper limb fracture patients is 4 - 6 days (5). In hip fracture patients, the average LOS is 11 days, with a significantly increased LOS seen in patients with concurrent upper limb fractures (6, 7). As the need for surgical intervention increases, so does the associated morbidity and mortality. Effective postoperative pain management is vital not only in preventing wound healing and rehabilitation complications but also in reducing the risk for chronic pain and long-term disability.

The goal of postoperative pain management is to relieve pain while minimizing side effects. A range of medications, most commonly opioids and local anesthetics, can be administered through various routes through two primary methods: Patient-controlled analgesia (PCA) and nurse-administered analgesia (NAA). The PCA allows patients to self-administer pain medication with an intravenous (IV), epidural, transdermal, or peripheral nerve catheter pump. By pressing a button linked to the pump, a patient can choose when to receive a preset dose of medication. The NAA relies on medical staff to assess a patient’s level of pain and administer medication accordingly. Both approaches are used to optimize postoperative pain management; however, PCA allows for more timely and individualized pain relief, giving patients a greater level of autonomy (8, 9).

The PCA and NAA have their advantages and disadvantages. The NAA provides the benefit of human interaction, is easy for patients to understand, and reduces the risk of medication tampering. However, NAA is time-consuming for medical staff and may lead to delays in pain relief due to competing responsibilities. There is also a risk of medication administration errors. The PCA can empower patients to manage their pain and reduce medical staff workload, but it comes with several risks, including pump malfunction, patients or staff having technical difficulties, and complications involving IV tubing, such as infection, kinks, and blockages. In some cases, malfunctioning PCA pumps can deliver medication at incorrect doses or intervals, posing additional safety concerns (10).

Previous studies have shown mixed outcomes regarding PCA's impact on LOS due to substantial variability across studied patient populations (11-13). In general, unnecessarily extended LOS are associated with higher rates of hospital-acquired complications, such as infections and falls, and higher costs for both patients and healthcare systems. Within postoperative orthopedic studies, findings on PCA’s impact on LOS have been mixed. A 305-patient retrospective observational study reported PCA unfavorably extended LOS in older patient groups who underwent total hip arthroplasty (14). In contrast, a retrospective cohort study showed that among 164 total knee arthroplasty (TKA) patients, PCA was found to shorten LOS when compared to NAA (15). Currently, there is limited research on directly comparing the effect of PCA versus NAA in LOS in patients undergoing limb fracture repair. Some studies have suggested that PCA use is associated with extensive surgeries, increased nausea, and prolonged time to rehabilitation, which may delay rehabilitation milestones and contribute to longer LOS (14, 16).

## 2. Objectives

To compare the LOS and pain scores among postoperative patients in a hospital system in the southern United States who underwent limb fracture repairs between 2019 and 2024, specifically evaluating outcomes in patients who received NAA versus those who received a combination of NAA and PCA. By analyzing differences in LOS and pain levels, this study can help determine whether different methods of pain management have an impact on patient recovery and clinical outcomes.

## 3. Methods

This study is a retrospective, non-interventional chart review that examines the LOS and pain scores among all postoperative patients between 18 and 75 years of age who underwent surgical repair of limb fractures between 2019 and 2024 in a hospital system in the southern United States. Data was collected retrospectively from the EPIC electronic medical record system, and the study was approved to be exempt from a local Institutional Review Board. All data was collected in compliance with all applicable institutional ethical guidelines and the health insurance portability and accountability act. Data was collected through the Northeast Georgia Health System (NGHS) clinical research data platform by a blinded graduate medical education data administrator. To ensure data integrity, ten percent of the data was validated by the data administrator and investigating co-resident.

Inclusion and exclusion criteria were developed to minimize the influence of confounding variables on the results. Included were patients between the ages of 18 - 75 with documented surgical repair of the following fractures: Wrist and hand (ICD-10-CM: S62), femur (S72), foot and toe excluding ankle (S92), forearm (S52), lower leg including ankle (S82), and shoulder and upper arm (S42). Excluded were patients who had nerve blocks for perioperative pain management (except when received on the day of surgery), patients with chronic pain requiring daily use of any analgesics for more than 30 days before surgery, and quadriplegic patients. 

Patients who met the inclusion criteria were divided into two groups: Non-PCA and PCA. The non-PCA group received NAA alone, whereas the PCA group received both NAA and PCA. This grouping approach is supported by literature highlighting the relevance of combining these methods in postoperative pain management (17, 18). Pooling the groups also enhances external validity and accounts for real-world clinical decision-making, which takes individual patient characteristics and relevant medical history into consideration. Consequently, the PCA group had both PCA and NAA involvement. The PCA group self-administered IV analgesics via PCA pumps in addition to receiving nurse-administered transdermal or intramuscular analgesics. The NAA group received transdermal, intramuscular, or IV analgesics exclusively via manual administration by nursing staff. Medications included in this study were morphine, oxycodone, hydromorphone, fentanyl, and acetaminophen. Collected data included demographic characteristics (e.g., age, gender), preoperative and postoperative pain scores measured by the Visual Analog Scale (VAS), and hospital LOS measured in days. Pain scores assessed using the VAS were documented by nursing staff throughout hospitalization in accordance with institutional protocols. Pain was recorded on a scale of 1 - 10 at two-hour intervals.

To address possible confounding bias in examining the relationship between PCA use and postoperative outcomes, propensity score matching was employed. Confounders and competing exposures that were identified through a directed acyclic graph (DAG) included age group, sex, race, ethnicity, primary language spoken, and mean preoperative pain score. Propensity scores were estimated with a logistic regression model, and a nearest neighbor matching algorithm was used to match 49 PCA patients to 49 non-PCA patients. Prior to matching, outliers' LOS were visually inspected, and two non-PCA patients with LOS values of 135 and 232 days were removed to reduce the influence of extreme values.

A negative binomial regression model was used to model the relationship between PCA use and LOS. The relationship between PCA use and postoperative pain scores was estimated with a normal regression model. Bootstrapping with 10,000 iterations was used to estimate model coefficients, standard errors, and confidence intervals (CIs). This approach was chosen to mitigate the risk of underestimating sample variation in small datasets, as relying solely on asymptotic results may be misleading (19). The bootstrap means were calculated as the point estimate for model coefficients. The 95% CI bounds were identified as the 2.5th and 97.5th quantiles of the bootstrap distribution. A Wald-like statistic was calculated for hypothesis testing to calculate a P-value. If P < 0.05, we rejected the null hypothesis of there being no association between PCA use and the outcome of interest. The incidence rate ratio (IRR) for LOS and the odds ratio (OR) for pain scores were calculated for the PCA cohort in both models, along with the respective 95% CIs to assess the strength and precision of the associations. All analyses were conducted using R (version 4.3.3) in the open-source RStudio IDE (Posit Software, PBC, Boston, MA, US).

## 4. Results

In this retrospective chart review, 3,127 patients were included in the non-PCA group, and 49 patients were included in the PCA group. Demographic data is shown in Table 1; overall, a greater proportion of patients in the study were over 45 years of age. In the non-PCA group, 76.3% were older than 45 years, with 42.8% falling in the 65 to 76-year age group. In comparison, 53% of patients in the PCA group were over the age of 45. Table 1 also shows demographic data after propensity score matching was used to match the 49 PCA patients with 49 non-PCA patients based on age group, sex, and preoperative pain score. While an age skew was noticed prior to matching, the age distribution post-matching was found to be balanced between patients younger and older than 45.

**Table 1.** Demographic Characteristics of Non-patient-controlled Analgesia and Patient-Controlled Analgesia Groups Before and After Propensity Matching ^a^

Variables	Non-PCA		PCA (N = 491)
	N = 31,271	N = 491 ^b^	
**Age (y)**			
18 - 24	193 (6.2)	4 (8.2)	6 (12.2)
25 - 34	262 (8.4)	8 (16.3)	8 (16.3)
35 - 44x	286 (9.1)	13 (26.5)	9 (18.4)
45 - 54	381 (12.2)	5 (10.2)	10 (20.4)
55 - 64	667 (21.3)	13 (26.5)	10 (20.4)
65 - 76	1,338 (42.8)	6 (12.2)	6 (12.2)
**Sex**			
Male	1,443 (46.1)	34 (69.4)	35 (71.4)
Female	1,684 (53.9)	15 (30.6)	14 (28.6)

Abbreviation: PCA, patient-controlled analgesia.

^a^ Values are expressed as No. (%).

^b^ Post-propensity matching.

Hospital course data were compared between the two groups. Both groups had similar preoperative pain scores, but the PCA group had slightly higher postoperative pain scores (Table 2). Table 2 also shows that after propensity matching, average preoperative pain scores between the non-PCA and PCA groups were similar at 5.64 and 5.60, respectively. Overall, the non-PCA group showed a greater reduction in pain between pre- and postoperative assessments when compared to the PCA group.

**Table 2.** Comparison of Hospital Course Data Between Non-patient-controlled Analgesia and Patient-Controlled Analgesia Groups Before and After Propensity Matching

Variables	Non-PCA		PCA
**Mean preoperative pain score**	5.20 (3.87, 6.50)	5.64 (4.20, 7.20) ^a^	5.60 (4.17, 7.22)
**Mean postoperative pain score**	4.21 (3.00, 5.27)	4.41 (3.18, 5.56) ^a^	4.92 (4.25, 5.68)
**Length of hospital stay (d)**	5 (3, 9)	5 (3, 8) ^a^	12 (7, 20)

Abbreviation: PCA, patient-controlled analgesia.

^a^ Post-propensity matching.

**Figure 1.**
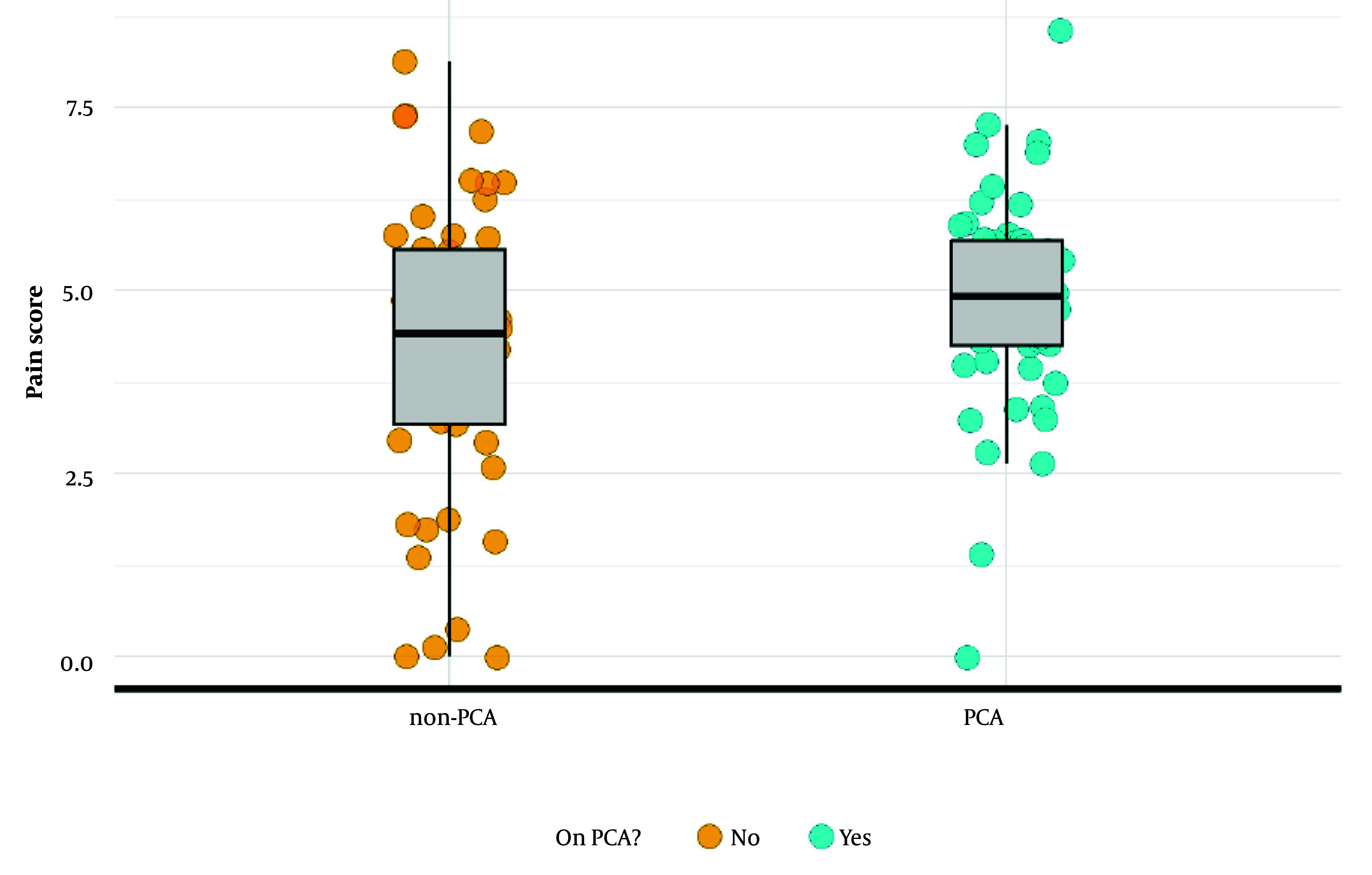
Postoperative pain score by patient-controlled analgesia (PCA) usage: Normal regression was used to model the relationship between PCA and post-surgery pain score. Patients using PCA exhibited a pain score two times greater [incidence rate ratio (IRR) = 2.004, 95% confidence interval (CI) 0.999 to 4.017, P = 0.046] than patients who did not receive PCA.

As postoperative pain was considered a possible contributing factor to longer LOS, further analysis was done and summarized in Table 3 and Figure 1. Without PCA, the baseline postoperative pain score is 4.190. Patients in the PCA group had higher mean postoperative pain scores (mean = 4.92) compared to the non-PCA group (mean = 4.41), with a mean difference of 0.51 points (P = 0.046). Although the CI includes 1, suggesting some uncertainty, the finding still leans toward a positive association between PCA use and higher pain scores.

**Table 3.** Modeling Results for Postoperative Pain Scores and Length of Stay

Variables; Coefficient	Beta Estimate ^a^	Beta SE	OR	IRR	95% CI	P-Value
**Postoperative pain scores**						
Intercept	4.190	0.276	-	-	-	-
PCA ^b^	0.695	0.348	2.004	-	(0.999, 4.017)	0.046
**LOS**						
Intercept	1.988	0.157	-	-	-	-
PCA	0.728	0.205	-	2.071	(1.375, 3.12)	< 0.001

Abbreviations: OR, odds ratio; IRR, incidence rate ratio; 95% CI, 95% confidence interval; PCA, patient-controlled analgesia; LOS, length of stay.

^a^ Log rate.

^b^ The PCA in comparison to non-PCA group.

**Figure 2.**
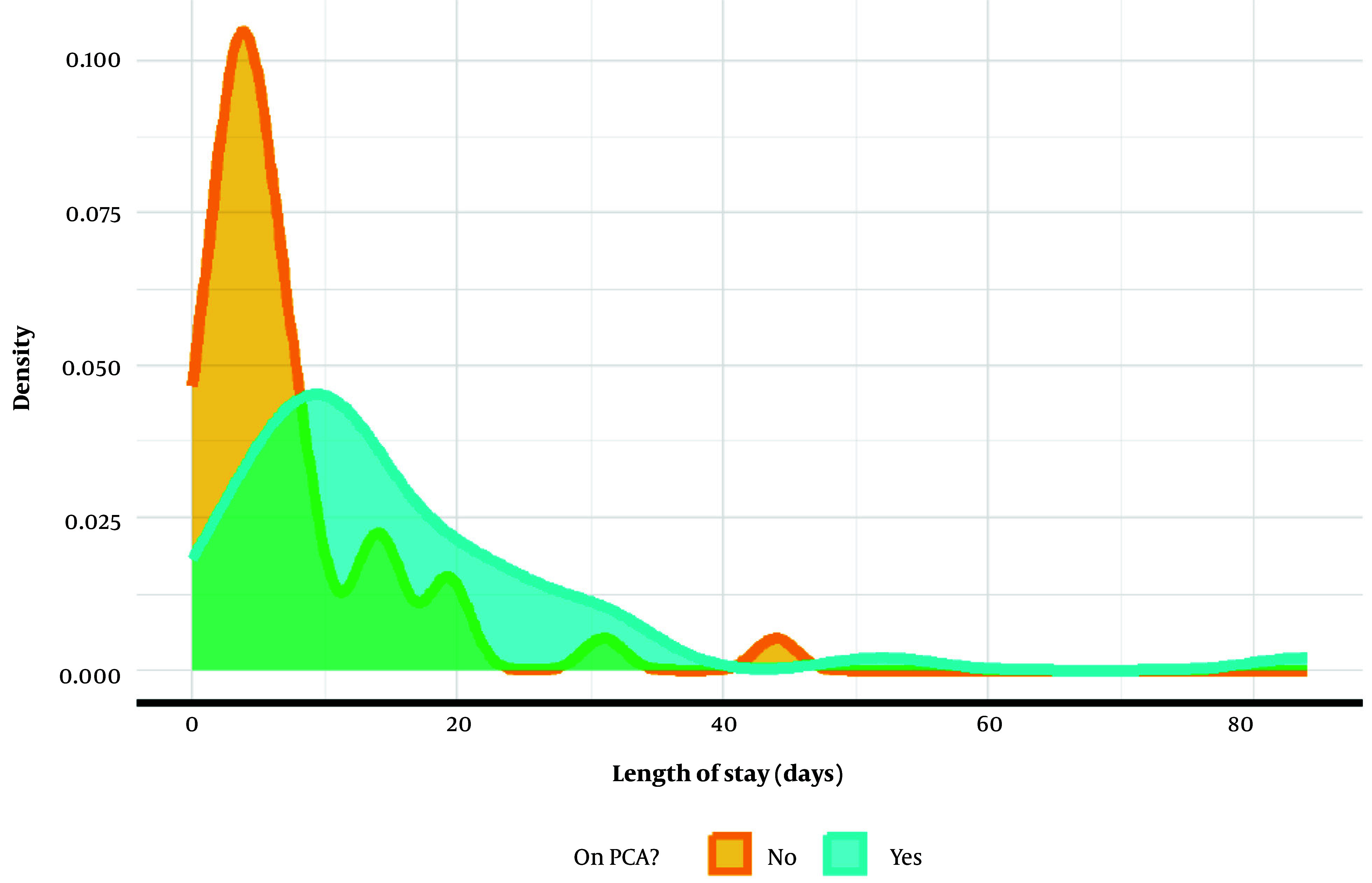
Length of stay (LOS) by patient-controlled analgesia (PCA) usage: Negative binomial regression was used to model the relationship between PCA and LOS. Bootstrapping was used to obtain model coefficient estimates, standard errors, and confidence intervals (CIs) through 10,000 iterations. Patients using PCA exhibited a LOS two times greater [incidence rate ratio (IRR) = 2.071, 95% CI 1.375 to 3.12, P < 0.001] than patients who did not receive PCA.

As shown in Table 3 and Figure 2, the PCA group had a significantly longer LOS, averaging 12 days compared to the non-PCA group’s average of 5 days. The PCA use was associated with a 2.1-fold increase in LOS duration, with an IRR of 2.071 (P < 0.001).

## 5. Discussion

Comparing PCA and NAA is crucial in understanding how different pain management strategies impact patient outcomes, particularly LOS and postoperative pain, which this study aimed to uncover. Following propensity score matching for age, sex, and preoperative pain scores, demographic characteristics were similar between the PCA and non-PCA groups. Bernabei et al. noted that older patients were more likely to be undertreated for pain (20). However, by matching for key demographic variables, our study minimized potential age-related biases.

In our study, the PCA group had higher postoperative pain scores and less postoperative pain relief compared to the non-PCA group. These findings align with a study conducted by Iddagoda et al., who also found worse pain control in their PCA group (14). Their study showed that PCA was linked to worse postoperative physical function, longer hospital stays, and higher odds of needing support at discharge. Similarly, our study found a longer average LOS in PCA patients, suggesting PCA may be less effective in pain control and recovery.

Increased pain scores among patients receiving PCA may be influenced by a complex interplay of factors such as chronic pain, psychological distress, cognitive impairment, and preexisting opioid tolerance (16, 21-23). Future research should aim to stratify patients based on these risk factors to develop personalized PCA strategies and explore adjunctive therapies.

When comparing our study with that of Cho et al., who conducted a prospective study on multimodal pain control versus PCA in rotator cuff repair, both studies highlight PCA's limited effectiveness in pain management (24). In their study, better pain control and earlier functional recovery were reflected in the multimodal pain control group, with significantly lower postoperative pain scores and fewer adverse effects reported. Duellman et al. compared the effects of multimodal preemptive analgesia versus PCA on postoperative outcomes following total joint arthroplasty (25). On average, the preemptive analgesia group had shorter LOS. The PCA patients consumed significantly more IV morphine and experienced a threefold increase in postoperative nausea. Additionally, PCA patients were twice as likely to miss rehabilitation therapy sessions and nearly twice as likely to be discharged to an extended care facility. While our study did not examine rehabilitation or post-discharge disposition, an increase in LOS would likely negatively impact both. Lahtinen et al. compared the use of PCA versus NAA in patients undergoing TKA (15). They found that opioid consumption and use of antiemetics during the first 24 hours post-surgery were similar in both groups. However, the PCA group had a significantly shorter LOS compared to the control group. In contrast, our study noted an increased LOS in patients who received PCA (15). However, our study focused on patients who underwent upper or lower limb fracture repair as opposed to patients who exclusively underwent TKA, as in this study. 

A Cochrane review by McNicol et al. evaluated the efficacy and safety of PCA versus non-PCA for postoperative pain management (17). The review included 49 studies with 3,412 participants. It found that PCA significantly reduced pain intensity compared to non-PCA, with patients having lower VAS scores at 24 and 48 hours after surgery. The PCA also led to higher opioid consumption and greater patient satisfaction. However, no significant difference in hospital LOS was observed between the two techniques. Overall, the review concluded that PCA is an effective alternative to non-PCA, although the quality of the evidence was rated as moderate to low. 

A study by Khan et al. evaluated whether PCA in a fast-track joint replacement program led to increased perioperative opioid consumption and longer LOS (18). This double-blind, randomized controlled trial involved 80 patients undergoing elective TKA. Patients were randomized into PCA and non-PCA groups. The results showed no significant differences between the two groups in terms of opioid consumption, LOS, pain scores, or opioid-related side effects. While many studies in the literature report greater pain relief and shorter hospital stays among patients receiving PCA, others highlight potential drawbacks, including higher opioid consumption, prolonged hospital stays, or decreased postoperative pain relief. Some research suggests that despite these concerns, the benefits of PCA — particularly enhanced patient autonomy and, in certain cases, reduced LOS — make it a viable alternative to NAA (8). However, several studies also found no significant differences in outcomes between PCA and NAA (12, 24). These findings underscore the importance of considering both the type of surgery and individual patient characteristics when evaluating the efficacy of PCA for postoperative pain management.

Our retrospective analysis suggests a notable association between PCA use and increased LOS as well as higher postoperative pain scores when compared to exclusive NAA use, findings that diverge from some prior research outcomes. These discrepancies underscore the complexity of postoperative pain management and the influence of patient and procedural variability. Future research with larger samples or the use of a Bayesian approach using empirical priors is recommended to better estimate these relationships and produce more robust conclusions. 

Current pain management protocols from prominent guidelines, such as those provided by the American Pain Society and the American Society of Anesthesiologists, emphasize the use of multimodal analgesia and individualized patient pain management strategies (16). The PCA is commonly recommended for its ability to allow patient autonomy and timely analgesic administration, improving patient satisfaction. However, its impact on clinical outcomes such as LOS and pain control compared to NAA remains less clear when considering results from McNicol et al., and Khan et al. (17, 18).

Despite our study's observational design limiting causative conclusions and the relatively small sample size, these findings contribute important preliminary evidence to an area of limited and unclear research. Considering the inconsistent findings in existing literature, our results highlight the necessity for larger-scale studies to rigorously evaluate the impact of PCA versus NAA on postoperative outcomes, ultimately refining clinical guidelines and enhancing patient care.

### 5.1. Conclusions

This retrospective analysis suggests an association between the use of PCA combined with NAA and longer LOS and higher postoperative pain scores when compared to exclusive NAA use in patients who underwent limb fracture repair. Older patients more often received pain medications than younger patients, regardless of the method of administration. Future studies should consider larger sample sizes or the use of a Bayesian approach to better estimate these relationships. The PCA may be applied based on clinical judgment when patients are expected to have higher pain scores and longer hospital stays. However, careful patient selection and monitoring are essential to optimize postoperative outcomes.

## Data Availability

The dataset presented in the study is available on request from the corresponding author during submission or after publication. The data are not publicly available due to privacy and HIPAA compliance.
